# Fosfomycin resistance in community-acquired urinary pathogens from Western Cape, South Africa

**DOI:** 10.4102/sajid.v37i1.321

**Published:** 2022-01-19

**Authors:** Lesedi B. Mosime, Mae Newton-Foot, Pieter Nel

**Affiliations:** 1 Division of Medical Microbiology, Department of Pathology, Faculty of Medicine and Health Sciences, Stellenbosch University, Cape Town, South Africa; 2 Department of Medical Microbiology, National Health Laboratory Service, Tygerberg Hospital, Cape Town, South Africa

**Keywords:** fosfomycin resistance, resistance mechanisms, prevalence, urinary tract infections, empiric therapy, Enterobacterales, Enterococcus spp., community-acquired UTI

## Abstract

Oral fosfomycin is commonly used to treat uncomplicated urinary tract infections (UTI) and whilst resistance has been reported in many healthcare facilities in South Africa, the current prevalence remains unknown. This study investigated the prevalence and mechanisms of fosfomycin resistance amongst urinary pathogens in the Western Cape, South Africa. Of the 200 isolates collected during the study period (2019–2020), seven (3.5%) were fosfomycin resistant. Mutations in the *glpT* and *uhpT* transporter genes were the most common mechanism of resistance detected. These findings support the ongoing use of fosfomycin as an empiric antibiotic choice for the treatment of community-acquired UTI in this setting.

## Introduction

Bacterial urinary tract infections (UTI) are common worldwide, affecting almost 150 million people annually.^1^ The most common causes of UTI are members of the Enterobacterales order as well as *Enterococcus* spp. Owing to increasing antibiotic resistance and side-effect concerns with the use of antibiotics such as ciprofloxacin and trimethoprim/sulfamethoxazole, fosfomycin has become an empiric antibiotic treatment option for uncomplicated UTI.^2^ Fosfomycin is active against both gram-positive and gram-negative pathogens, including *Enterococcus* spp., *Escherichia coli, Klebsiella* spp., *Enterobacter* spp., and *Proteus mirabilis*. In addition to nitrofurantoin and intramuscular gentamicin, fosfomycin is recommended by the South African Department of Health as a first-line agent for the treatment of uncomplicated UTI in women.

Fosfomycin resistance primarily occurs by modification of the antibiotic target because of mutations in the *murA* gene, which reduces the affinity between the *murA* protein and the fosfomycin molecule. Fosfomycin resistance may also be as a result of the inactivation of the hexose phosphate (UhpT) and glycerol-3-phosphate (GlpT) transport systems, thereby decreasing uptake of the antibiotic.^3^ Another mechanism of fosfomycin resistance involves the production of enzymes such as FosA, FosB, FosC and FosX, encoded by the *fos* genes, which inactivate fosfomycin by cleaving the oxirane ring.^3^ Of these, FosA enzymes are most frequently reported and are common in Enterobacterales.^4^

Reports of fosfomycin resistant *E. coli* are increasing worldwide, with resistance rates of approximately 3.2% reported in Europe, Asia and the United States (US).^5^ Similarly, fosfomycin resistance rates of up to 16% have been reported in vancomycin-resistant *Enterococcus faecium* in North America.^6^ In South Africa, fosfomycin resistance rates of 4.3% – 4.5% have been reported in UTI isolates from Gauteng with resistance in major Enterobacterales pathogens (*E. coli, Klebsiella* spp., *P. mirabilis* and *Enterobacter* spp.) ranging between 2.0% and 8.0% and resistance rates in *Enterococcus* spp. reaching 2.0%.^7^

At the Tygerberg Hospital National Health Laboratory Service (NHLS) Medical Microbiology diagnostic laboratory (Cape Town, Western Cape, South Africa), fosfomycin susceptibility testing is routinely performed on all Enterobacterales isolates, except *E. coli*, when resistance to other commonly prescribed oral antibiotics is noted. Despite the occasional detection of fosfomycin resistance in our setting, the current prevalence and underlying mechanisms of fosfomycin resistance remain unknown. This study aimed to determine the prevalence of fosfomycin resistance amongst community-acquired urinary pathogens from the Western Cape and to describe the mechanisms of resistance in these isolates.

## Methods and materials

### Study design

This was a laboratory-based descriptive study performed at the Division of Medical Microbiology at Tygerberg Hospital in the Western Cape of South Africa, which serves approximately 2.6 million people. Over a period of four months (October 2019 – January 2020), 200 isolates were cultured from urine samples received from antenatal clinics. Pregnant women visiting antenatal clinics routinely submit urine samples for medical health screening. Any organisms isolated from these samples were considered to be representative of community carriage.

Isolation, identification and antimicrobial susceptibility testing (AST) of urinary pathogens were performed as part of routine diagnostic procedures in the laboratory. This included urine culture on UriSelect™ selective chromogenic agar medium (Diagnostic Media Production, Green Point, South Africa) for isolation and differentiation of urinary pathogens. Identification of organisms and routine AST were performed on the automated VITEK^®^ 2 (bioMérieux, France) platform. The VITEK^®^ 2 provides AST results in the form of estimated minimum inhibitory concentrations (MIC) for multiple organism/antimicrobial combinations including gram-positive and gram-negative bacteria.^8^ Organism susceptibility was interpreted according to the 2019 Clinical and Laboratory Standards Institute (CLSI) guidelines.

### Fosfomycin susceptibility testing

Fosfomycin susceptibility of all isolates was determined by the Kirby-Bauer disc diffusion method using a fosfomycin disc (200 µg) containing 50 µg glucose-6-phosphate (G-6-P) (Mast Group Ltd, United Kingdom) on Mueller-Hinton (MH-Sens) agar plates (Diagnostic Media Production, Green Point, South Africa). For fosfomycin resistant isolates, the fosfomycin MICs were determined by gradient diffusion with fosfomycin E-test^®^ strips (0.064 µg/mL – 1024 µg/mL) (Liofilchem, Italy). Strips were placed on MH-Sens agar plates inoculated with a 0.5 McFarland standard bacterial suspension, and incubated at 37 °C for 16 h in the presence of 5% carbon dioxide. All zone sizes were measured from the disc to the closest colony growth and E-tests® were read as per the manufacturer’s guidelines. No mutant colonies grew within the E-test® ellipses. The disc diffusion and MIC results were interpreted according to the CLSI 2019 guidelines and reported as either susceptible (zone of inhibition [ZOI] ≥ 16 mm, MIC ≤ 64 µg/ml), intermediate (ZOI 13–15 mm, MIC 128 µg/ml), or resistant (ZOI ≤ 12 mm, MIC ≥ 256 µg/ml). The CLSI guidelines only provide breakpoints for *E. coli* amongst Enterobacterales and *E. faecalis* amongst *Enterococcus* spp.; therefore, these breakpoints were inferred for all Enterobacterales and *Enterococcus* spp., respectively.

### Molecular detection of fosfomycin resistance genes

DNA was extracted from fosfomycin resistant isolates using a crude heat-freeze DNA extraction method. All isolates were screened for *fosA*1-7 by polymerase chain reaction (PCR) amplification using previously described primers.^5,9^ Isolates positive for *fosA*3/4 and *fosA*5/6 were obtained from an in-house isolate collection and used as positive controls following Sanger sequencing to confirm target specificity. In the absence of a control for *fosA*7, the *fosA7* PCR product was confirmed by Sanger sequencing and used as a positive control for subsequent PCR reactions. There were no controls available for *fosA*1/2. Fosfomycin resistant *E. coli* and *K. pneumoniae* were also subjected to PCR and Sanger sequencing to characterise mutations in the chromosomal genes *murA, glpT* and *uhpT,* using previously described primers.^10,11,12^ All PCR reactions were performed using KAPA Taq ReadyMix (KAPA Biosystems, US). Primer sequences and PCR reaction conditions are described in Online Appendix 1, Table 1. The PCR products were visualised by agarose gel electrophoresis and Sanger sequencing was performed at Inqaba Biotech^TM^ (South Africa). *E. coli* and *K. pneumoniae* chromosomal gene sequences were aligned to *E. coli* strain K-12 substr. MG1655 (ref: NC_000913.3) or *K. pneumoniae* subsp. *pneumoniae* HS11286 (ref: NC_016845.1), respectively, using the BioEdit Sequence Alignment Editor 20 to identify potential mutations in *murA, glpT* and *uhpT*.

**TABLE 1:** Mutations detected in fosfomycin resistant *E. coli* and *K. pneumoniae* isolates.

Organism	*fosA*	Sequence variants		
		*murA*	*glpT*	*uhpT*
*E. coli* (CA3)	ND	ND	*Thr348Asn*	**Deletion** (68 bp)†
*E. coli* (CA4)	*fosA*7	ND	*Glu374Ala*	ND
*E. coli* (CA5)	ND	ND	*Gly415Asp; Asn450Thr*	**Deletion** (96 bp)‡; *Thr425Ala*
*K. pneumoniae* (CA6)	ND	*Ser148Asn; Thr206Lys; Ser210Thr*	ND	**Deletion** (38 bp)§; *Val434Ile*

Note: Italics denotes mutations that have not been described previously; Bold denotes deletions in *uhpT* that have previously been shown to confer resistance.

ND, not detected; bp, base pairs.

† , Nucleotide 985–1053;

‡ , Nucleotide 1293–1389;

§ , Nucleotide 1117–1155.

### Ethical considerations

Because of the inclusion of secondary non-human data in this study, ethical approval for waiver of consent was obtained from the Stellenbosch University Health Research Ethics Committee (reference number: S19/08/168).

## Results

### Study samples and species distribution

**FIGURE 1:**
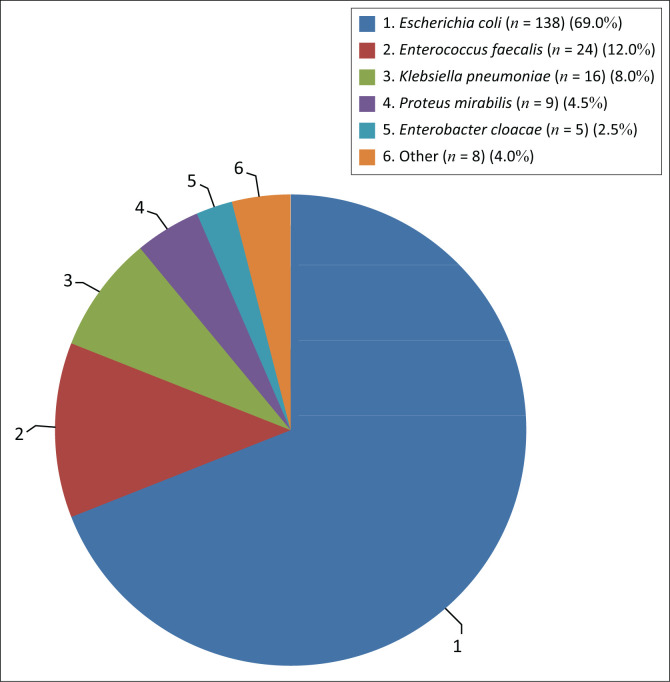
Species distribution of 200 urinary isolates collected from patients at antenatal clinics in the Western Cape. Organisms classified as ‘other’ include *Klebsiella oxytoca* and *Klebsiella aerogenes* (*n* = 2, 1% each), and *Citrobacter freundii, Citrobacter koseri*, undefined *Enterococcus* spp. and *Morganella morganii* (0.5%, *n* = 1 each).

Of the 200 isolates cultured from urine samples received from antenatal clinics, *E. coli* was the most predominant species (*n* = 138; 69%), followed by *E. faecalis* (*n* = 24; 12%) (see Figure 1).

### Fosfomycin susceptibility

Seven (3.5%) of the 200 isolates were resistant to fosfomycin: 3/138 (2.2%) *E. coli,* 2/5 (40%) *E. cloacae,* 1/16 (6.3%) *K. pneumoniae* and 1/10 (10%) *P. mirabilis*. One isolate (*E. cloacae*) had an MIC of 512 µg/ml and the rest of the isolates had MICs of > 1024 µg/ml, which were all interpreted as fosfomycin resistant according to the CLSI 2019 criteria. All fosfomycin resistant isolates were susceptible to ciprofloxacin and trimethoprim/sulfamethoxazole and most were intermediate (3/7, 43%) or susceptible (3/7, 43%) to nitrofurantoin. Fosfomycin resistance was not detected amongst the *Enterococcus* spp. isolates.

### Fosfomycin resistance mechanisms

All fosfomycin resistant isolates were screened for the *fosA*1-7 genes, however, only *fosA*7 was detected in a single *E. coli* isolate. Fosfomycin resistant *E. coli* (*n* = 3) and *K. pneumoniae* (*n* = 1) isolates were screened for chromosomal mutations in the *murA, glpT* and *uhpT* genes. Mutations in *murA* were only identified in the *K. pneumoniae* isolate, and these had not been previously described (see Table 1). The fosfomycin resistant *E. coli* isolates harboured previously undescribed mutations in the *glpT* gene (see Table 1), as well as three mutations, Leu297Phe, Glu443Gln and Gln444Glu, which have been reported to have no impact on fosfomycin susceptibility.^11^ The *K. pneumoniae* isolate, as well as the two *E. coli* isolates which did not harbour *fosA*, contained deletions of multiple nucleotides in the *uhpT* gene. Additional previously undescribed mutations in *uhpT* were identified in one *E. coli* (CA5) and the *K. pneumoniae* isolate.

## Discussion

The fosfomycin resistance rate amongst community-acquired urinary pathogens from the Western Cape of South Africa was low at 3.5% (7/200); with no fosfomycin resistance detected amongst the *Enterococcus* spp. isolates. Amongst the *E. coli*, fosfomycin resistance was detected in 2.2% of isolates, which is similar to the recently reported 2% resistance in hospital-acquired UTI *E. coli* isolates in Johannesburg.^7^ *E coli* is not routinely tested for fosfomycin resistance at the Tygerberg Hospital NHLS Medical Microbiology diagnostic laboratory because of the presumed low prevalence of resistance and these findings support this practise.

*fosA*7 was only detected in one fosfomycin resistant isolate, which suggests that *fosA* activity is not a common cause of resistance amongst community-acquired urinary pathogens in this population. The deletions observed in *uhpT* in two *E. coli* and one *K. pneumoniae* isolate are likely to confer resistance as *uhpT* deletions have been reported to be the most common mutations involved in gene inactivation in both clinical and *in vitro* generated fosfomycin resistant isolates.^13^ This should be further investigated in follow-up functionality studies.

There was growth of single colonies within the ZOI, making disc diffusion interpretation difficult and operator dependant. Elliot et al.^4^ suggested that the growth of single colonies within the ZOI may be caused by the presence of chromosomal *fosA* genes rather than chromosomal mutations. Whole genome sequencing of scattered colonies’ genomes may indicate the common genes that are harboured by these colonies and could improve the interpretation of diffusion susceptibility testing methods in the laboratory.

None of the *E. coli* isolates in this study harboured mutations in the *murA* gene, but all three had mutations identified in the *glpT* gene and two had additional mutations identified in the *uhpT* transporter genes (Table 1). Mutations in the *murA* gene are common in most fosfomycin resistant organisms except *E. coli*, where they have been associated with a high biological cost.^14^ The role of the previously reported Thr348Asn mutation, detected in the *glpT* gene of one of the *E. coli* isolates, in fosfomycin resistance has not been established.^14^ The Glu374Ala, Gly415Asp and Asn450Thr mutations detected in *glpT* in *E. coli* have not been described before, therefore their role in fosfomycin resistance remains unknown. Other mutations such as Leu297Phe, Glu443Gln and Gln444Glu, that have been previously described, were detected in the *glpT* gene of three *E. coli* isolates, but they have previously been proven not to confer resistance.^11^ There were no positive controls for *fosA*1/2 genes, making it possible that these genes were missed during PCR detection. The small fosfomycin resistant sample set and the general lack of correlation between genetic mechanisms of resistance and phenotypic expression, complicated the interpretation of our results. Furthermore, we could only base our findings on the selected resistance genes and mutations investigated in this study.

Future studies on this sample set could use whole genome sequencing to describe other potential fosfomycin resistance mechanisms, including the detection of other *fos* genes and mutations in genes such as *ptsI, uhpA* and *cyaA,* that have also been previously reported to contribute to fosfomycin resistance. Functional characterisation of previously uncharacterised mutations should also be performed to confirm their role in fosfomycin resistance.

## Conclusion

The prevalence of fosfomycin resistance in community acquired UTI in the Western Cape of South Africa remains low (3.5%). The most common mechanism of fosfomycin resistance was deletions in the transporter gene *uhpT*. This study served as a reminder of the challenges related to fosfomycin susceptibility testing and highlighted the need to improve these testing methods. Our findings support the ongoing use of fosfomycin as an empiric choice for the treatment of community acquired UTI. Close clinical follow-up of patients is however essential when treating UTIs caused by pathogens other than *E. coli* and *E. faecalis*.
